# Polymer/Carbon Nanotube Based Nanocomposites for Photovoltaic Application: Functionalization, Structural, and Optical Properties

**DOI:** 10.3390/polym14061093

**Published:** 2022-03-09

**Authors:** Boubaker Zaidi, Nejmeddine Smida, Mohammed G. Althobaiti, Atheer G. Aldajani, Saif D. Almdhaibri

**Affiliations:** 1 Department of Physics, College of Science and Humanities, Shaqra University, Dawadmi 11911, Saudi Arabia; s442481193@std.su.edu.sa (A.G.A.); s442500416@std.su.edu.sa (S.D.A.); 2 Laboratoire de Synthèse Asymétrique et Ingénierie Moléculaire de Matériaux Organiques Pour L’électronique Organique LR 18ES19, Department of Physics, Faculty of Science, University of Monastir, Monastir 5019, Tunisia; 3 Department of Chemistry, College of Science and Humanities, Shaqra University, AlQuwaiiyah 19257, Saudi Arabia; nejm.smida@su.edu.sa; 4 Laboratory of Interfaces and Advanced Materials, Faculty of Science, University of Monastir, Monastir 5019, Tunisia; 5 Department of Physics, Faculty of Science, Taif University, Taif 21974, Saudi Arabia; m.althobaiti@tu.edu.sa

**Keywords:** PVK, hexylthiophene, PANI, nanocomposite, photovoltaic cells, DFT

## Abstract

We present a systematic review of nanostructured organic materials, including synthesis methods, functionalization, and applications. First, we report the chemical and physical procedures used for preparing the polymer/carbon nanotube composites described in the literature over the last decade. We compare the properties of different polymer-based prototypes of organic nanocomposites functionalized with carbon nanotubes. Theoretical and experimental vibrational investigations provide evidence of the molecular structure describing the interaction between both components, showing that the allowed amount of carbon nanotubes and their dispersion states differ across polymers. Moreover, the nature of the solvent used in the preparation has a significant impact on the dispersion process. The integration of these materials in photovoltaic applications is discussed, where the impact of nanoparticles is evidenced through the correlation between experimental analyses and theoretical approaches based on density functional theory. Alterations in optical properties, evaluated from the absorption and luminescence process, are coherent with the solar spectrum, and a good distribution of donor/acceptor interpenetration was observed. In all cases, it was demonstrated that the performance improvement is physically related to the charge transfer from the organic matrix to the nanoparticles.

## 1. Introduction

Scientific communities are continuously exploring innovative properties of π-conjugated conducting polymers, used as active layers in various organic electronic devices [1,2,3,4]. Hence, diverse molecular engineering procedures have been reported, such as doping conjugated polymers with conductive elements to ameliorate their conductivities [5,6]. Compared to inorganic semiconductors, these materials offer good flexibility and easier processing, leading to easier modulation of electronic, optical, and mechanical properties [7,8,9,10,11,12,13]. Handicaps related to fragility and a lower operating lifetime can be overcome by adding a small amount of carbon nanotubes (CNTs) [14,15]. Recently, Subramanyam et al. demonstrated that the addition of CNTs helps achieve an increase in lifetime by 67% in an open atmosphere [16]. According to Francis et al., CNTs also improve charge transport and provide good thermal stability, allowing π-conjugated conducting polymers to be good candidates for electrical, thermal, sensing, and actuating applications [17]. If CNTs are well dispersed and the exciton diffusion length is sufficient, the configuration leads to charge and energy transfer in the organic matrix [18,19,20]. This transfer is a major parameter for good photovoltaic conversion. Hence, the separation process of electron–hole pairs should dominate if the diffusion length is sufficiently higher than the mean distances between nanoparticles. The resulting interpenetrating (bulk P–N hetero-nanojunctions) network results in a good overlap between the optical absorption (OA) spectral region and the solar spectrum [21]. Indeed, the decrease in the optical gap involves additional absorption features in the visible spectrum and leads to a higher charge separation and good collection efficiencies [22,23,24,25]. Hybrid materials, including inexpensive flexible polymers and their nanostructures, represent the next generation of photovoltaic solar cells [26]. Continuous progress in and improvement of fabrication methods is a major focus of research [27]. The aim is to ensure a uniform distribution and arrangement of nanoparticles in the polymer matrix. The performance of organic photovoltaic solar cells is comparable to that of monocrystalline silicon, where PCE reaches 24% [16].

**Scheme 1:**
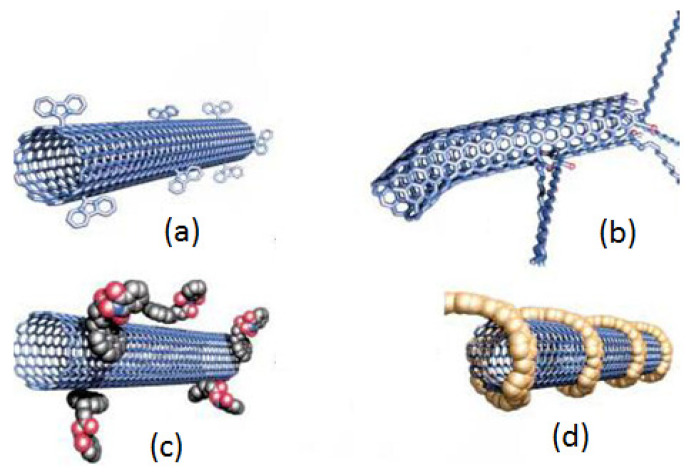
Interaction between CNTs and functional molecules covalently (**a**,**b**) and noncovalently (**c**,**d**) [39].

CNTs exist in a bundled form in the solid state. The dispersion process requires mechanical or vibrational energy higher than van der Waals cohesive forces [28]. The direct mixing methods are based on mechanical agitation or vibrational forces using homogenizers with different velocities [29,30]. CNTs with better homogeneity and alignment can be achieved by applying to the nanocomposite an ultrasound wave and centrifugal forces of appropriate power for an appropriate amount of time [31,32]. Chemical or physical functionalization, through covalent or noncovalent bonding between CNTs and diverse active molecules, has also been reported [33,34]. For chemical functionalization of the CNT surface (Scheme 1a,b), the bonding can be via defect groups or by side wall bonding, as well as by other special reactive groups, such as lithium alkyl nitrenes and fluorine radicals [35,36,37]. These chemical modifications facilitate the grafting of other functional groups and give rise to covalent bonding with the polymers/molecules [38]. However, noncovalent functionalization can be hexahedral (Scheme 1c,d) or endohedral [39].

**Scheme 2:**
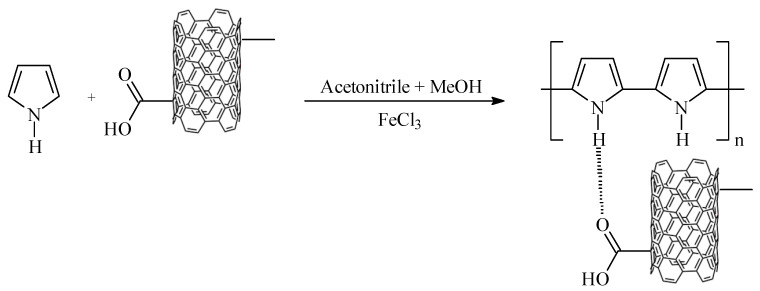
Interaction between polypyrrole and multiwalled CNTs.

For a polymer moiety, the polymerization process can be carried out after chemical functionalization, where first the monomers are linked to the tube and then the polymerization takes place [40,41]. If the direct mixing of polymers or in situ polymerized fragments with CNTs results in noncovalent linking, the process is called physical functionalization (Scheme 2) [42,43,44,45,46].

The dispersion process of nanoparticles is often of short duration. These nanoparticles have the tendency to agglomerate on the surface. To separate aggregates, the obtained solution should be exposed to ultrasonic baths and centrifuge forces with controlled frequencies and power [47]. In this work, we present various methods used over the last two decades for nanocomposite elaboration for a specific class of polymeric materials and their impact on the amelioration of photovoltaic properties. The study is restricted to polymer materials containing nitrogen in the aliphatic or cyclic sequences, such as poly(N-vinyl-carbazole) (PVK), poly(3-hexylthiophene (P3HT), and polyaniline (PANI). We report the results of our investigations in the last decade of the different polymeric nanostructures based on a large variety of polymers. As indicated for nanostructured materials, photoexcitations are followed by the establishment of charge transfer. The electronic structure modification, including the highest occupied molecular orbital (HOMO) and the lowest unoccupied molecular orbital (LUMO) levels, localized states, their energies, and the corresponding skin depth, should be evaluated in order to reduce injection barriers. Density functional theory (DFT) calculations are additional alternatives applied to a justified model structure to support experimental characterizations and physical interpretations [48,49,50,51,52,53]. Properties of organic materials that are experimentally inaccessible are in general described by DFT-based quantum calculations [50,53,54].

## 2. Organic Nanocomposite Synthesis and Characterization

### 2.1. CNT Treatment and Dispersion

CNTs (single-walled or multiwalled forms) need some treatment before functionalization with organic materials. Purification can be ensured either chemically, using a selective oxidation processor, or physically, based on filtration and/or centrifugation [55,56,57,58]. Metallic and semiconducting nanotubes can be separated by gradient-of-density ultracentrifugation techniques. The difference in their diameters is also exploited in the separation process [59,60]. Selective interaction with a conducting polymer is an alternative procedure that is efficient for separation in an acidic medium [61].

**Figure 1:**
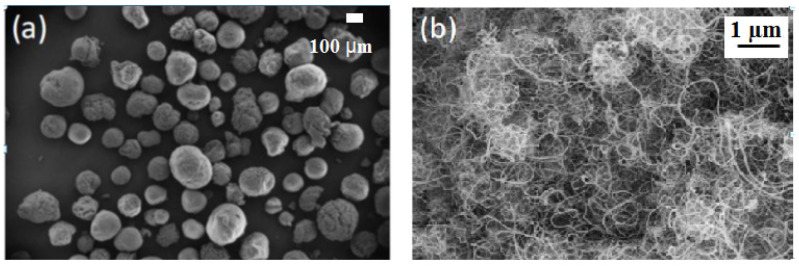
Structure of the bundled CNT (**a**); a zoom-in photo of the aggregates (**b**).

Good dispersion (homogeneous distribution) of CNTs in the polymer matrix is a major step that influences their properties [62]. CNTs largely exist in the bundled form due to strong van der Waals cohesive forces. It is difficult to achieve the isolated form, and the functionalization process leads to aggregate formation (Figure 1). Concerning the composite preparation, purified single-walled carbon nanotubes (SWCNTs) are dispersed in the same solvent with a polymer for an appropriate amount of time. The transitory dispersed phase of SWCNTs is immediately added in the desired amount to the polymer solution. Then, the sonication process and centrifuge forces are applied.

### 2.2. Materials and Methods

Composite solutions were deposited at room temperature with nearly uniform thickness on silica substrates for OA measurements. However, glass slides were used for FTIR and photoluminescence (PL) measurements. All substrates were already cleaned with deionized water and ethanol in an ultrasonic bath. All samples thus obtained were introduced into a Pyrex tube and heated under vacuum at a moderate temperature based on the thermal stability of the resulting material. Infrared absorption measurements were recorded with a resolution of 2 cm^−1^. The OA spectra were recorded in the absorption mode with the wavelength varying from 200 to 2000 nm. For the PL experiment, depending on the material emission spectral range, different excitation wavelengths were used (280, 325, and 540 nm). Ultrafast time-resolved PL measurements were carried out with the regenerative amplified femtosecond laser system. The time-resolved emission spectra were spatiotemporally detected with a high-dynamic-range Hamamatsu C7700 streak camera with a temporal resolution <20 ps. The PL intensity and decay times were simulated with two coupled exponential decays. The TEM images were observed after adding liquid nitrogen to the Cu grid and then were taken at temperatures varying from 100 to 104 K. Theoretical data were procured from quantum calculations based on density functional theory (DFT). The modeling structure was fully optimized using the most popular Becke’s three-parameter hybrid functional, B_3_ [63], with the basis set 6-31G(d). Calculations applying to the proposed modeling structures were carried out with nonlocal correlation of Lee–Yang–Parr (LYP), abbreviated as the B_3_LYP method. Theoretical OA spectra were calculated using the time-dependent density functional theory (TD-DFT) method with the 6-31G(d) basis.

### 2.3. Alignment and Orientation of CNTs

**Figure 2:**
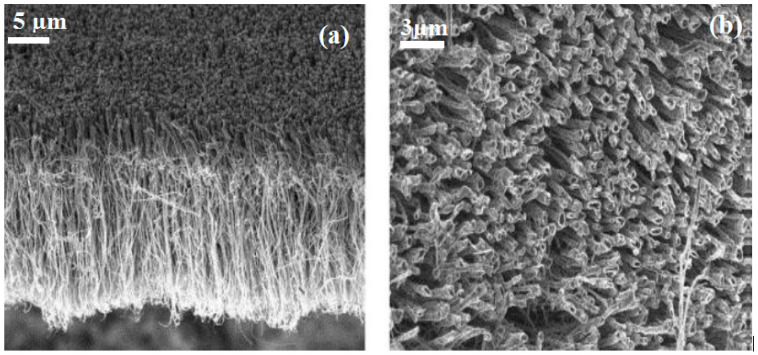
TEM pictures illustrating CNT orientation observed in (**a**) the lateral direction and (**b**) the longitudinal direction [69].

CNT orientation can be accomplished in solution during or after the polymer synthesis process. Chemical vapor deposition synthesis of SWCNTs results in a massive growth of superdense and vertically aligned nanotubes with enhanced catalytic activity of catalysts (Figure 2) [64]. CNTs can also be aligned under a magnetic or an electric field during the growth step, but some dynamic structural changes can occur due to the complexity of the process [65,66,67,68].

**Scheme 3:**
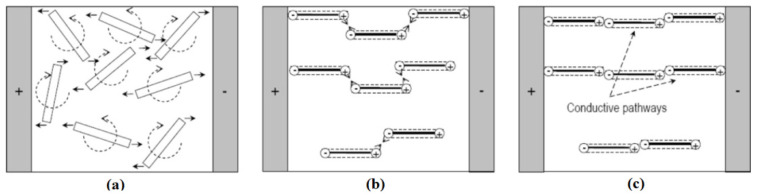
Network formation: (**a**) orientation, (**b**) interaction between oriented CNTs, and (**c**) the resultant network [76].

After CNT dispersion, the application of external electrical or magnetic perturbations allows the orientation of CNTs in the preferred direction [70,71,72,73,74,75]. This network formation results in three steps: the orientation of nanotubes, interaction, and network establishment (Scheme 3). Due to dipolar interaction, the aligned CNT configuration leads to tail-to-tail or head-to-head connections.

In both cases, the oriented CNTs permit the reinforcing of optical and electronic transport properties in the preferred direction. This process helps enhance the performances of the electronic and optical devices. Moreover, annealing at moderate temperatures leads to a good repartition of CNTs into the organic polymeric matrix, which results in higher photovoltaic performances [77,78,79].

## 3. Applications of Organic Nanocomposites for Photovoltaic Cells

Organic solar cells represent new alternatives to those based on inorganic semiconductors [16]. Organic materials are characterized by flexibility, lightness, low cost, and easy processing. However, their intrinsic charge carrier mobility is lower (at max 10^−1^ cm^2^ V^−1^ s^−1^) compared to that of their homolog inorganic semiconductors, where it can reach 900 cm^2^ V^−1^ s^−1^ for a single silicon crystal. Otherwise, handicaps related to lower mobility and fragility can be partially resolved when adding CNTs or phenyl C61 butyric acid methyl ester (PCBM) because carbon structures exhibit better mobility and rigidity than silicon [80]. Therefore, adding a small quantity of CNTs (single-walled or multiwalled forms) may improve the mechanical properties and lead to a good compatibility with the solar spectrum (UV–visible range). If, additionally, there is coherence between diffusion length (~20 nm) and the distances between CNTs, the separation process of electron–hole pairs will be improved. In fact, when adding CNTs, the entire network will be regular nano-P–N junctions, which represent photogeneration sites in all points. The charges transferred from the organic matrix to CNTs may be simultaneously collected and carried by CNTs. In the literature, the open-circuit current for some elaborate photovoltaic devices is in the range of a few tens of milliamperes per square centimeter [81,82,83,84]. This nanometric configuration is different from the conventional inorganic P–N photovoltaic cell. Indeed, for an inorganic device, a single junction separates the P and N regions, while in the nanometric interpenetrating network, junctions are distributed in the entire bulk, giving rise to more photoexcitations, leading to higher efficiency.

For organic photovoltaic conversion, efficiency started with values lower than 1%. However, the conversion efficiency was considerably improved beginning from the year 2000, reaching 2.5% and then 3.6% when PCBM was inserted in the organic matrix [85,86]. The relatively high conversion efficiency is obtained with a double heterostructure of fullerene (C_60_) and copper phthalocyanine [87]. In 2011, a new record of 5% was attained using hybrid CNT/polymer materials and then ameliorated to reach 12% [10]. Compared to a silicon monocrystal, the power conversion efficiency (PCE) remains lower, but the easy processing and low cost make the material attractive [10]. The actual records were achieved by Cui et al. in 2020 and Subramanyam et al. in 2021, reaching, respectively, 17% and 24%, comparable to the PCE of porous and crystalline silicon [16,88].

## 4. Nanocomposites of Oligo-N-vinyl-carbazole/Single- and Multiwalled CNTs

Infrared, Raman, OA, and PL spectroscopies were used to describe the interaction between OVK and SWCNTs in polar and apolar solvents. The effect of the solvents’ polarities on the dispersion process, i.e., repartition of SWCNTs on the organic matrix and its relationship with optical and vibrational properties, is reported. The observed changes in characteristics and their interpretations are discussed according to the literature [89,90,91,92]. Experimental results are correlated to those theoretically obtained from DFT; the functional and the method are described elsewhere [63,93,94,95].

### 4.1. Vibrational Properties

**Figure 3:**
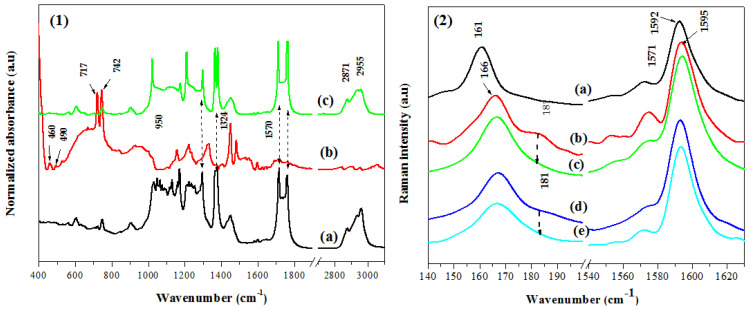
(**1**) Infrared spectra of the OVK/SWCNT composite dispersed in a chloroform solvent (**a**) and a chlorobenzene solvent (**b**) not annealed and (**c**) annealed at 333 K. (**2**) Raman radial and tangential modes: (**a**) SWCNTs; OVK/SWCNT in chloroform in the not-annealed (**b**) and annealed (**c**) states; OVK/SWCNT in chlorobenzene in the not-annealed (**d**) and annealed (**e**) states.

OVK/SWCNT composites were prepared at various concentrations in different solvents, such as chloroform and chlorobenzene, and were annealed to ameliorate the dispersion process [21,96]. FTIR spectra depicted in Figure 3 reveal that the most relevant vibrational modes of native OVK and SWCNTs are found in the composite spectrum. Moreover, in both cases, adding SWCNTs leads to some significant changes. Some bands with a relatively higher intensity ascribed to OVK vinyl groups disappear, which is strictly related to the linking between SWCNTs and OVK. This grafting is spontaneously established in the case of chloroform. However, when dispersing in chlorobenzene, certain spectra can be recorded after annealing treatment at 393 K (as in the case of nanocomposite dispersed in chloroform). Indeed, a better modification of the spectrum is clearly seen after annealing, and both spectra (a) and (c) present higher similarity. In both cases, the spectra reveal the appearance of a new feature at 1297 cm^−1^ that indicates the covalent interaction between organic OVK matrix and CNTs, as described for electrosynthesized PVK/SWCNTs [97].

Raman analyses (Figure 3(2)) reveal the presence of SWCNT bands, but those of OVK are severely attenuated. Radial and tangential modes depend on the used solvents and thermal treatment, which reflect the dispersion state of CNTs in the organic matrix (decrease in the band attributed to the bundled form at 181 cm^−1^).

Both radial mode bands at 161 and 181 cm^−1^ are associated, respectively, with isolated and bundled SWCNTs [98,99]. The mean diameter of the tube can be evaluated from the empirical relation and varies from 1.34 to 1.38 nm [96,100]. The shift in radial mode toward higher wavenumbers has been interpreted as a consequence of inserting organic molecules into bundled SWCNTs. The disappearance of radial mode (bundled CNTs) in the case of the chlorobenzene solvent leads to the conclusion that the polar solvent leads to more dispersed nanotubes. The change from 2D to 3D symmetry is illustrated by the tangential mode narrowing and the decrease in intensity for the mode at 1571 cm^−1^ [101]. The spectrum of annealed states reveals shifting and band intensity modifications, promoting a better functionalization process.

### 4.2. Changes in Optical Properties

**Figure 4:**
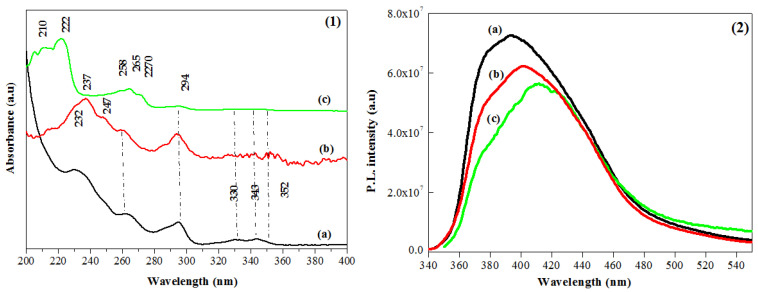
Changes in OA (**1**) and PL (**2**) spectra. Before SWCNT addition (**a**) and after SWCNT addition in chloroform (**b**) and in chlorobenzene (**c**).

The most relevant information on the excitation and de-excitation process can be described by the complementarity of OA and PL measurements (Figure 4).

Absorption spectra are affected by adding SWCNTs. For both solvents (chloroform and chlorobenzene), we see a redshift and a new band apparition, implying an interaction between both components. When chloroform is the solvent, in the spectral region of 400 to 2000 nm, there is a gradual decrease in the absorbance, similar to the case of other polymers when functionalized with SWCNTs [21,102]. Bands that appear in this spectral region (λ > 400 nm) correspond to the optical transitions for semiconducting SWCNTs. In chlorobenzene, the hypsochromic shift of the blue-side transition implies a decrease in the oligomer chain length and/or a decrease in the interchain interaction due to the good dispersion of SWCNTs in the organic matrix [81].

**Figure 5:**
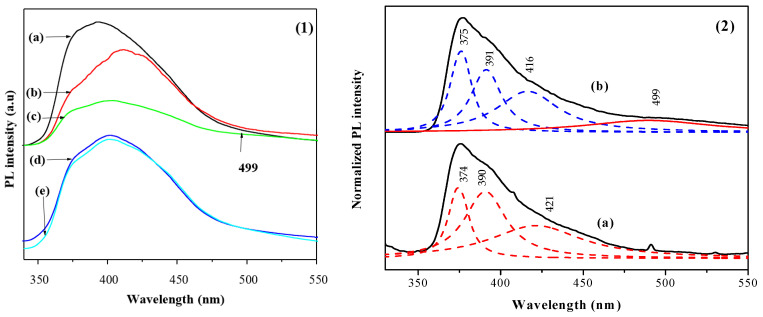
(**1**) Stationary PL spectra of (**a**) OVK and of nanocomposite in chloroform (**b**) not annealed and (**c**) annealed at 333 K and in chlorobenzene (**d**) not annealed and (**e**) annealed at 333 K. (**2**) PL spectra at T = 87 K of both annealed samples (**a**) OVK and with the OVK/SWCNT composite (**b**).

Chlorobenzene as a solvent quenches PL intensity after SWCNT insertion more efficiently (Figure 4(2)). This result is in good agreement with Raman results supporting good SWCNT dispersion. Indeed, photoexcitations are followed by a separation process rather than a recombination, because of which the observed PL is quenched [89,103,104]. We think that the photoexcitation produced in the OVK matrix can reach SWCNTs where the dissociation process is more preponderant rather than the recombination process. After annealing, the quenching effect becomes more pronounced and a new feature emission is created at around 499 nm (2.48 eV) when chloroform is the solvent (Figure 5(1)). As at a lower temperature (T = 87 K) and for both annealed samples (with and without SWCNTs), this weak emission band appears only in the case of the composite. It is, therefore, the consequence of the insertion of SWCNTs (Figure 5(2)).

### 4.3. Interaction between SWCNTs and an Organic Matrix

**Scheme 4:**
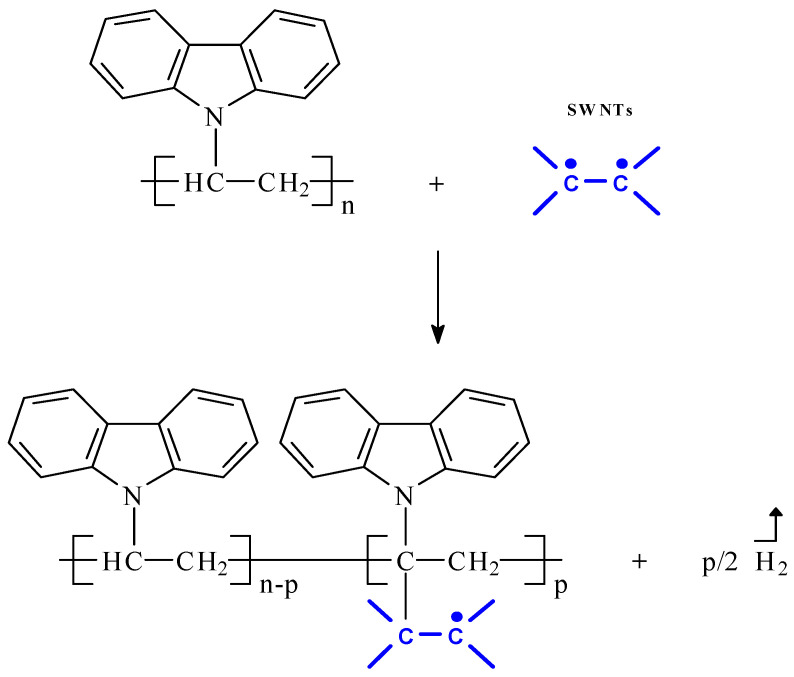
Grafting mechanism of OVK and CNTs.

The correlation of experimental and theoretical results evidences the covalent functionalization process between SWCNTs and oligo-N-vinyl carbazole [21]. The process does not require any thermal treatment when the dispersion is in chloroform as the solvent. The most affected chemical environments are vinylidene groups because, we assume, grafting occurs between these groups and the side wall (Scheme 4), similar to the case of PVK/MWCNT and for PVK/C60 composites [98,101]. However, after annealing, the similarity of both spectra supports the same grafting process.

**Figure 6:**
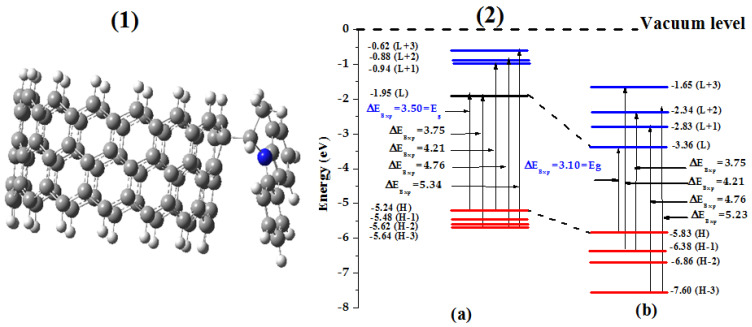
(**1**) Nanocomposite modeling structure used for DFT calculation and (**2**) change in the electronic structure from OVK (**a**) to the nanocomposite (**b**). H: HOMO; L: LUMO; Exp: experimental.

The proposed grafting mechanism, confirmed in our previous work, is consolidated by theoretical vibrational frequencies obtained either for OVK or for OVK/SWCNT composites [21]. First, experimental results are reproduced to confirm the modeling structures of primary materials. Then, the most probable grafting sites with higher reactivity are supported by force constant calculations in both neutral and oxidized states. In the resulting model structure of the nanocomposite, therefore, OVK is grafted on the side walls via vinylidene groups (Figure 6(1)).

Based on the above modeling structure, DFT has also been calculated with the B3LYP/3-21G* method to carry out changes in the electronic structure. All the theoretical changes are depicted in Figure 6(2), which are in good accordance with experimental absorption transitions.

### 4.4. Structural Properties in Relationship with Transient Photoluminescence

**Figure 7:**
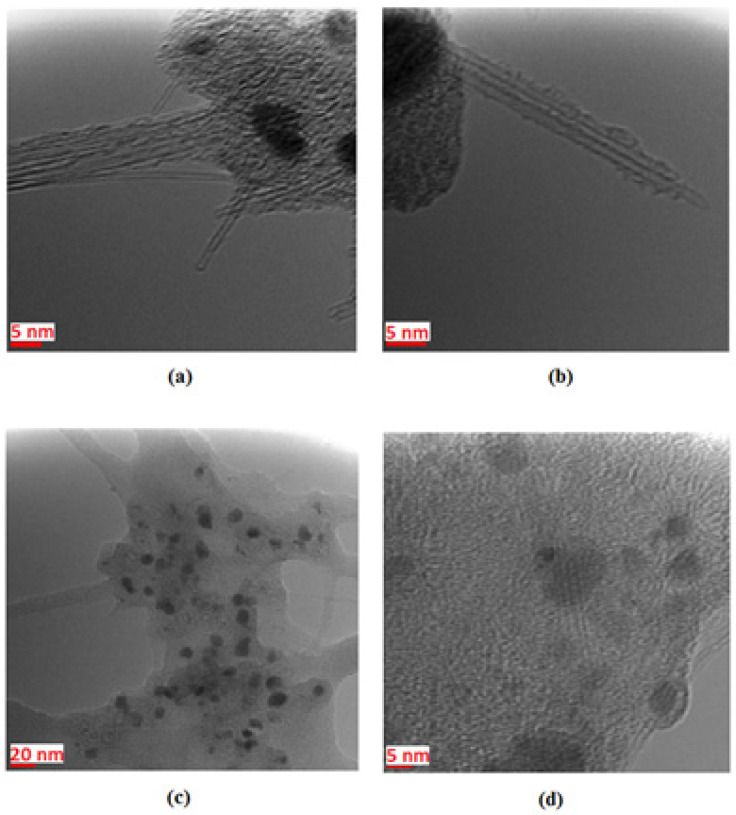
TEM pictures of OVK/SWCNT composites dispersed in chloroform (**a**,**b**) and in chlorobenzene (**c**,**d**) [18].

Transmission electron microscope analyses were carried out to have an idea about the SWCNT distribution and arrangement (Figure 7) [18].

When chloroform is the solvent, bundled and isolated SWCNTs are detected, but with a lower concentration for the isolated form, supporting a good SWCNT dispersion process. Figure 7c shows a good uniformity and orientation of SWCNTs having an interdistance of nearly 10 nm, probably due to the thermal treatment. In chlorobenzene (Figure 7d), this distance varies from 3 to 20 nm and the structure contains bundled SWCNTs connected by individual ones. In fact, these structural differences can influence the dynamical properties of the excited states.

Results of PL decay (obtained by pumping at 330 and probing at 390 nm) show a decrease in lifetimes and imply exciton diffusion to the SWCNTs [18,105,106] (Table 1).

**Figure 8:**
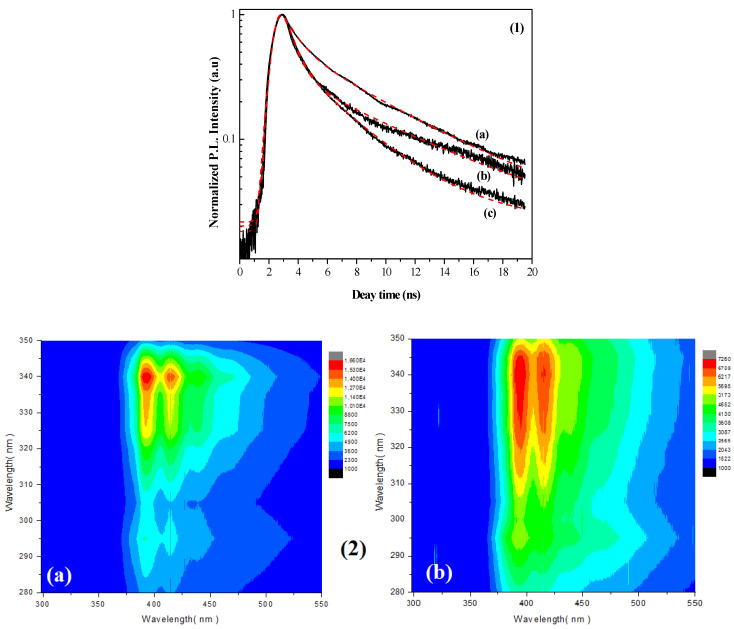
(**1**) PL decay OVK (**a**), OVK/SWCNTs in chloroform (**b**), and OVK/SWCNTs in chlorobenzene (**c**) (―: experimental; (….): theoretical). (**2**) PL cartography of OVK/SWCNTs in chloroform (**a**) and in chlorobenzene (**b**).

The existence of two populations weighing P_1_ and P_2_, having lifetimes of τ_1_ and τ_2_, and having a mean lifetime τ_m_ has been interpreted to be because of shorter and longer segments, respectively. A similar decay evolution was reported in the case of other polymers functionalized with various nanoparticles [107,108], supporting a charge transfer between the organic matrix and SWCNTs [105,109]. Lifetimes evaluated from Figure 8(1) are shorter than those of PVK, which is coherent with the fact that faster decay is a signature of shorter segments [110,111]. The photoexcitations that occur in the organic matrix will diffuse and be transferred into the SWCNTs, and the electron–hole pairs will be separated due to the internal electric field created by hetero-nanojunctions [81,83]. The obtained decay times are coherent with PL cartography (Figure 8(2)), in which we show a more intense luminescence when chlorobenzene is the solvent. Indeed, a faster lifetime results in the condensation of excitations in the radiative pathways.

## 5. Properties of PVK-3HT/SWCNT Nanocomposites

To reach a better dispersion process with a higher CNT concentration and to obtain good optical properties, another matrix containing PVK was synthetized [29,112]. Hexylthiophene, known by a large optical spectrum, was bridged with PVK, where the resulting matrix is called PVK-3HT. PVK-3HT/SWCNT nanocomposites were studied by FTIR and Raman spectroscopy, where the SWCNT weight concentration varied from 0% to 60%. It was shown that in all cases, SWCNTs are bundled and isolated. However, the D-band and the G-band, situated at 1250 and 1595 cm^−1^, respectively, are nearly the same in all cases, similarly to the case of purified nanotubes.

### 5.1. Vibrational Study

**Figure 9:**
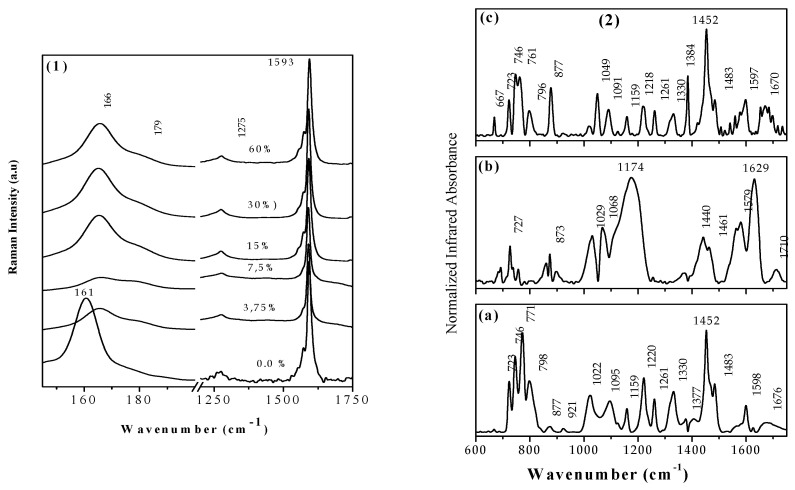
(**1**) Raman spectra of PVK-HT/SWCNTs at different CNT weight concentrations (0%, 3.75%, 7.5%, 15%, 30%, and 60%). (**2**) FTIR spectra of PVK-HT (**a**), SWCNTs (**b**), and PVK-HT/SWCNT composite (60%) (**c**).

On adding SWCNTs to PVK-3HT, there is no apparent change in the band positions and shapes of both compounds. However, the band intensities change and the appearance of new vibrational features at 1049 and 1384 cm^−1^ (Figure 9(2)) lead to the conclusion that there is an interaction between both components.

### 5.2. Optical Properties Changes

**Figure 10:**
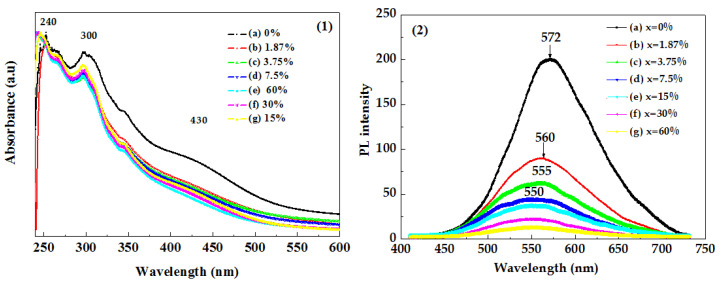
(**1**) OA and (**2**) PL spectra in chloroform solvent of pristine PVK-HeT (**a**) and those of PVK-HeT/SWCNT composites at various SWCNT weight concentrations: (**b**) 1.87%, (**c**) 3.75%, (**d**) 7.5%, (**e**) 15%, (**f**) 30%, and (**g**) 60%.

UV–visible absorption analyses (Figure 10(1)) demonstrate that PVK-HT absorbs in the large spectral domain. The absorbance shape observed in the spectral domain ranging from 240 to 300 nm is the signature of the grafting of bicarbazole units as a part of the resulting copolymer [113]. The extension of the absorbance in the visible range indicates extended conjugated fragments of P3HT in the obtained copolymer [113,114]. On adding SWCNTs, no apparent change is observed for λ < 400. However, the band near 430 nm decreases and blue-shifts, which agrees well with the hypothesis of the diminution of interchain interaction and/or the decrease in the π-conjugation length [115].

The continuous PL quenching (Figure 10(2)) shows that the addition of CNTs has a direct impact on the excited-state dynamics of the prepared composite (exciting at 400 nm using a seep range of 1 ns) [111]. As in the case of the OVK/SWCNT composite, we think that the exciton diffusion length becomes progressively higher than the distance between neighboring nanotubes. In this case, the separation process is the most dominant behavior that can occur.

**Figure 11:**
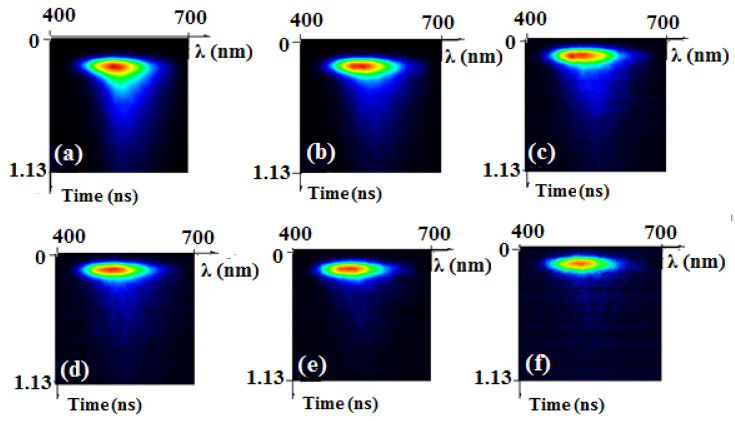
Contour plot of transient PL for PVK-HTs functionalized with different SWCNT concentrations: (**a**) 0%, (**b**) 1.87%, (**c**) 7.5%, (**d**) 15%, (**e**) 30%, and (**f**) 60%.

These results are in full accordance with the progressive decrease in emitted energy intensity as a function of time from 0 to 1 ns obtained in 3D maps (Figure 11).

**Figure 12:**
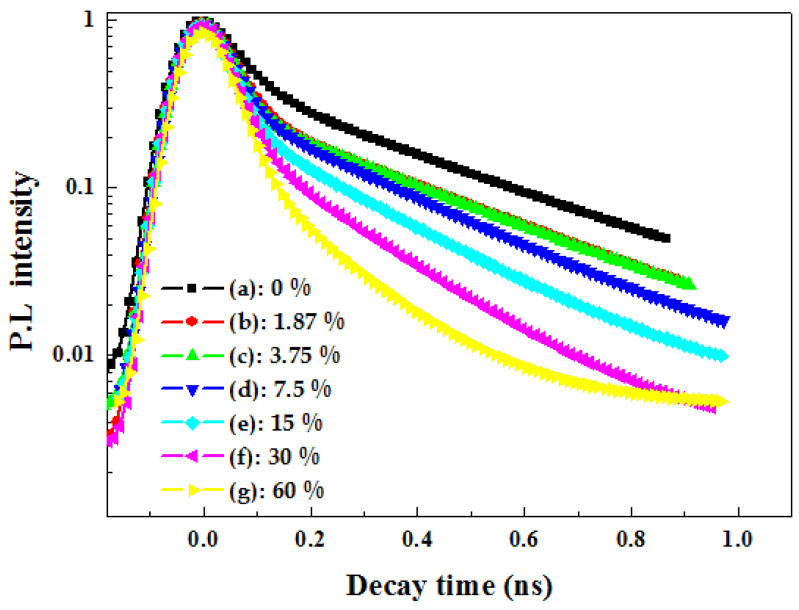
Time-resolved PL decays of PVK-HTs functionalized with SWCNTs at different concentrations: (**a**) 0%, (**b**) x = 1.87%, (**c**) 3.75%, (**d**) 7.5%, (**e**) 15%, (**f**) 30%, and (**g**) 60%.

Time-resolved PL spectra are presented in Figure 12. A progressively faster decay time is obtained when increasing the SWCNT weight concentration, as in the case of the PPV/SWCNT composite [111]. This behavior confirms that SWCNTs play a role in charge separation after exciton diffusion. Indeed, a shorter lifetime is an indicator of the decrease in the distance traveled by the exciton. The increase in the SWCNT concentration will, therefore, progressively reduce these distances. If these distances become lower than the intrinsic diffusion length, charge transfer from the organic matrix to CNTs can occur. This transfer leads to a separation of electron–hole pairs rather than their recombination, because of which there is a progressive quenching of stationary PL. 

## 6. PANI/SWCNT Nanocomposites

Following the same procedure, PANI, known by a number of oxidation degrees, is also functionalized with CNTs. The starting material, the emeraldine base (PANIEB purity 99.99%; macromolecular mass Mn > 15,000), is first doped with sulfonic acid and then functionalized with SWCNTs in the dimethylformamide (DMF) solvent [116]. The sulfonic acid doping procedure used in this case is described by MacDiarmid et al. [117].

### 6.1. Vibrational Study

**Figure 13:**
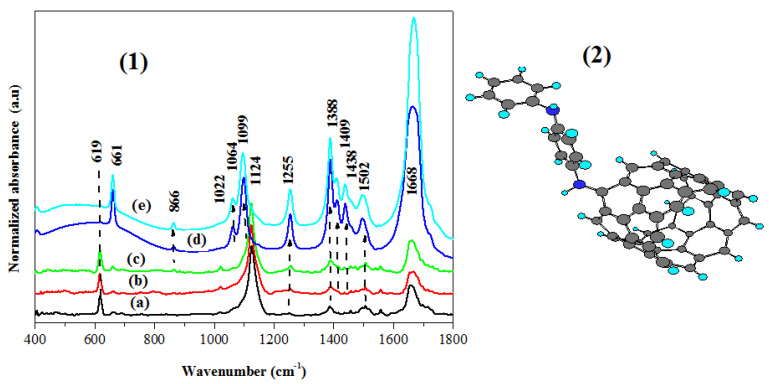
(**1**) FTIR spectra of PANI/SWCNT nanocomposites with various SWCNT concentrations: (**a**) 0%, (**b**) 0.71%, (**c**) 1.16%, (**d**) 2.10%, and (**e**) 5.00%. (**2**) Model structure of the nanocomposite used for DFT calculation [19].

The normalized FTIR spectra of Figure 13(1), when referring to the already published vibrational studies, reveal that concentrations lower than 2.10% cannot significantly induce apparent changes [118,119]. Starting from 2.1%, the linking of both components is evidenced by the appearance of new features at 1064, 1255, 1409, and 1438 cm^−1^. Then, the apparent shift of both modes at 1124 and 619 cm^−1^, the disappearance of the band at 1022 cm^−1^, and the enhancement of bands in the range of 1350–1700 cm^−1^ reflect the new molecular arrangement induced by covalent bonding [120,121].

The weak band located at 1022 cm^−1^ ascribed to the C–H deformation disappears, and a new band appears at 1062 cm^−1^. The bands situated in the range from 1350 to 1700 cm^−1^ show a better intensity increase, supporting the increase in aromaticity in the resulting compound. Two other new bands appear, one at 1409 cm^−1^ and one at 1438 cm^−1^, and confirm covalent binding between both components via a C–N link [121].

A theoretical calculation including bond length, atomic charge, and spin density lead to the conclusion that nitrogen atoms are the most probable grafting sites, as in the case of similar woks [19,119]. In Figure 13(2), we illustrate the model structure that has been used for DFT calculation. Noncovalent bonding can also be present as a consequence of the strong van der Walls forces that inhibit the homogeneous dispersion process [122,123].

### 6.2. Optical Property Changes

**Figure 14:**
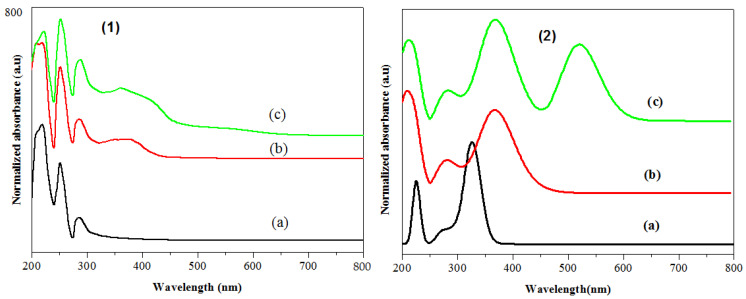
OA spectra of PANI in various states: (**1**) experimental and (**2**) theoretical; (**a**) neutral, (**b**) doped, and (**c**) the nanocomposite.

The modeling structure proposed above has been supported by theoretical calculation of OA spectra (Figure 14). The same transitions are found either experimentally or theoretically, for which it is noted that there is a diminution of band gap after either doping or adding SWCNTs. The energy gap of PANI in its neutral state is 3.75 eV; it decreases when doped and reaches 2.63 eV. A new band appears at −535 nm on adding SWCNTs, and the gap is 1.7 eV. This new band corresponds to the charge transfer where electron–hole pairs appear in the organic matrix, and then it is transferred to the SWCNTs by the diffusion process, leading to enhanced photovoltaic performances [18,96,124].

### 6.3. Electronic Study

**Figure 15:**
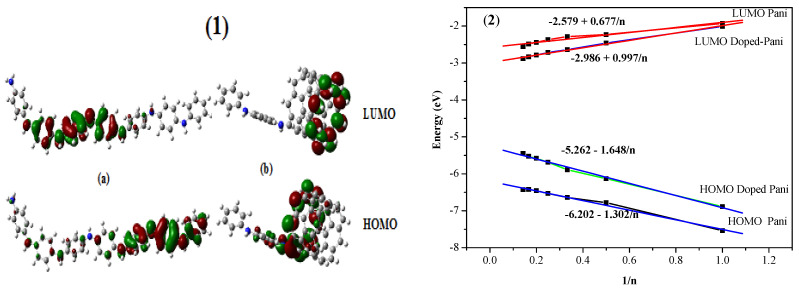
(**1**) HOMO and LUMO orbitals: doped PANI (**a**) and nanocomposite (**b**). (**2**) Effect of chain length on the HOMO–LUMO energy levels of PANI in pristine and doped states.

The study on SWCNT concentration leads to the conclusion that localized states are created in the band gap with the energy of 1.62 eV and with the band width of almost 80 meV (Urbach energy) [121]. This donor–acceptor charge transfer is more elucidated by contour plots of molecular orbitals (MOs), shown in Figure 15(1) [125]. HOMO and LUMO state densities are, respectively, localized on donor and acceptor moieties [126].

The ability of the resulting material to improve photovoltaic conversion is checked from its electronic structure (Figure 15(2)). Indeed, HOMO–LUMO energy levels are calculated from the optimization of model structures of pristine and doped states for different chain lengths [127].

The HOMO–LUMO energy levels for an infinite chain length can, therefore, be evidenced following Aleman et al. [127], where the theoretical energy gaps are 3.58 and 2.31 eV, respectively, for neutral and doped states. Other parameters can be evaluated, such as the PCE and the open-circuit voltage (V_OC_). From the individual theoretical electronic structures (donor and acceptor), PCE can be evaluated using the Scharber diagram. Otherwise, the V_OC_ can be calculated according to Equation (1) based on the HOMO and LUMO energy levels of, respectively, donor and acceptor materials [128,129,130,131]:

(1) $$V_{\mathrm{OC}}\left(V\right)=\frac{1}{e}\left|E{\left(\mathrm{HOMO}\right)}^{\mathrm{donor}}\left(\mathrm{eV}\right)\right|-\left|E{\left(\mathrm{LUMO}\right)}^{\mathrm{acceptor}}\left(\mathrm{eV}\right)\right|-0.3\;\left(V\right)$$

**Figure 16:**
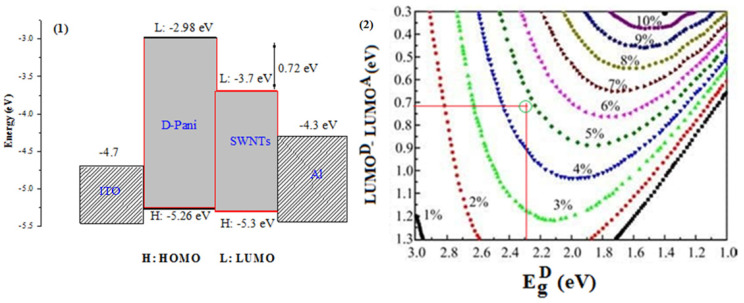
(**1**) Electrode choice for the photovoltaic device. (**2**) Estimation of PCE from the Scharber diagram [19].

The choice of metal electrode is strictly based on the values of HOMO and LUMO energy levels for doped PANI and CNTs. It is found that Al and ITO are the most appropriate for the device architecture (Figure 16(1)). Using Equation (1), the V_oc_ is estimated at nearly 1.25 V, and using the Scharber diagram, PCE is estimated at 4–5% (Figure 16(2)). This is in good accordance with our previous PL results showing both the quenching effect and the total absence of luminescence in the visible region [116]. 

### 6.4. Effect of Annealing Treatment on the PANI–SWCNT Composite

**Figure 17:**
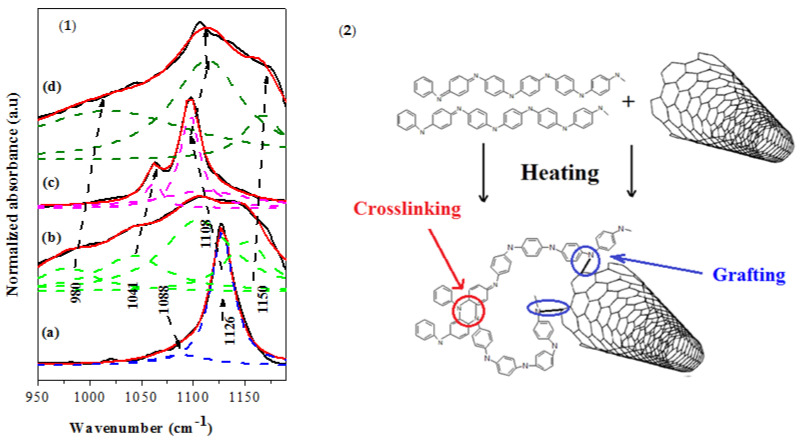
(**1**) Splitting of the FTIR band at 1126 cm^−1^ under annealing: pure PANI (**a**), annealed PANI (**b**), not-annealed composite (**c**), and annealed composite (**d**). (**2**) Structure of the nanocomposite obtained after annealing at 393 K.

Vibrational and optical behaviors of the hybrid material after annealing were analyzed in a previous study [132]. Interpretation of changes in FTIR spectra has shown the grafting and self-cross-linking of PANI moieties [133,134]. More details of the molecular structure are evidenced by the splitting of the band at 1126 cm^−1^ after annealing (Figure 17(1)). These chemical environments could not be the consequence of CNT insertion (the same line shape appears with and without CNTs). These additional bands are typically coherent with self-cross-linking (Figure 17(2)), such as benzene ring para- and ortho-link, to nitrogen atoms [132].

**Figure 18:**
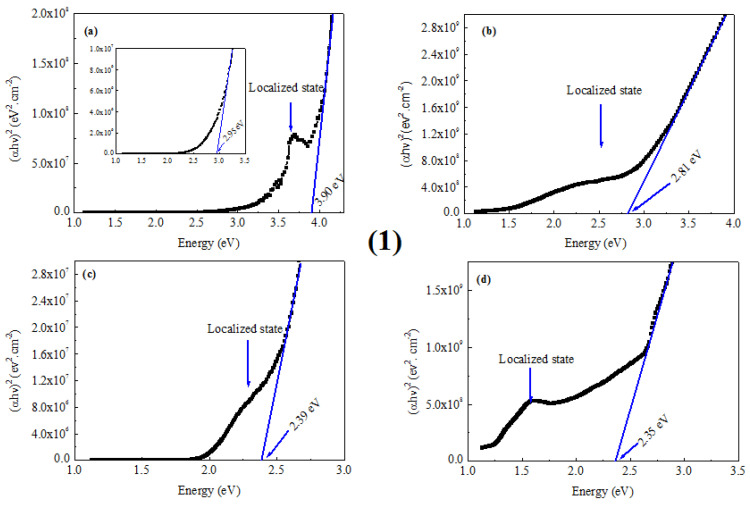

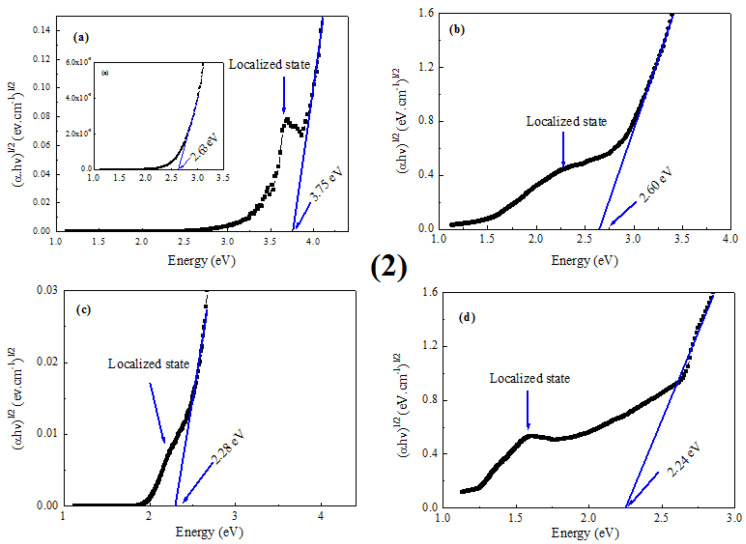
Evaluation of direct (**1**) and indirect (**2**) band gap for doped PANI in the not-annealed (**a**) and annealed (**b**) states and those of the doped PANI/SWCNT composite in the not-annealed (**c**) and annealed (**d**) states. The inset of (**a**) shows the threshold energy of localized states created by doping.

After annealing at 393 K, new absorption bands are created at λ_max_ = 570 nm and at λ_max_ = 792 nm, respectively, for doped and functionalized states [132]. These new optical features support the presence of a quinoidal ring and agree well with the self-cross-linking process [135,136]. The direct and indirect band gaps are evaluated from absorbance coefficient data using the Tauc method [137,138]. The direct and indirect energy gaps (Eg) are calculated from the linear dependence of the plot of (αhν)^1/n^ versus energy (Figure 18) [139,140,141].

As previously reported, both direct and indirect transition processes dominate optical transitions since direct and indirect energy gaps are nearly similar and the variation ($\Delta E=(E_{\mathrm{Dir}}-E_{\mathrm{Ind}})/E_{\mathrm{Ind}})$ is in the range from 4% to 8% [138]. The value of the transition probability index *n* has been also calculated to evaluate the weight of optical transition mode (values vary from 0.82 to 1.24).

**Figure 19:**
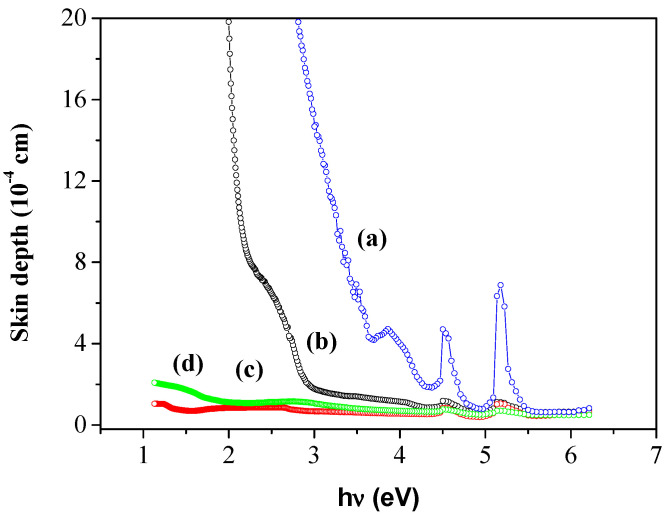
Skin depths of doped PANI: not annealed (**a**) and annealed (**b**); skin depths of doped PANI functionalized with CNTs: not annealed (**c**) and annealed (**d**).

The skin depth variation as a function of incident photon energy is one of the most relevant parameters reflecting the importance of adding CNTs (Figure 19).

When SWCNTs are added, the skin depth decreases; it decreases further after annealing, indicating the reduction in the transparency volume. This result demonstrates that the addition of CNTs leads to a better interfacial strength, as proved in a previous thermal study [142]. At a higher photon energy, the two picks of skin depth are the signature of plasma frequency, resulting from overlapping between the frequency of electron oscillation and the incident electromagnetic radiation (the wave is stagnant).

**Figure 20:**
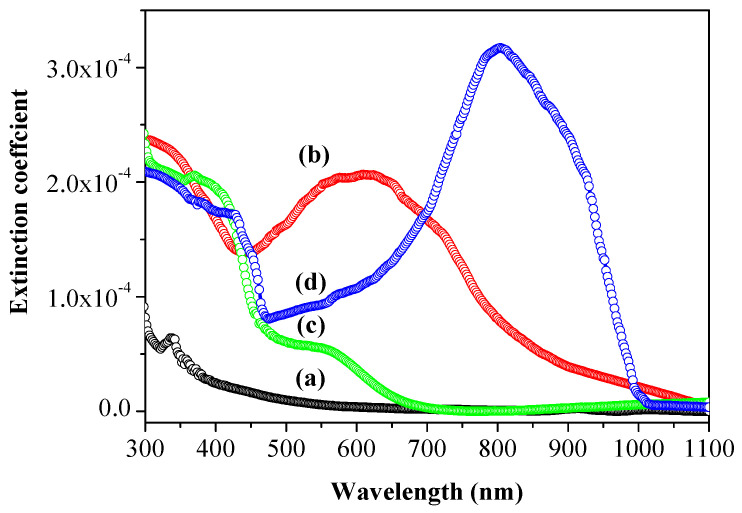
Extinction coefficients of doped PANI: not annealed (**a**) and annealed (**b**). Extinction coefficients of doped PANI functionalized with CNTs: not annealed (**c**) and annealed (**d**).

The variation in the extinction coefficient versus energy is another indicator of the change in optical properties after the addition of CNTs in both annealed and not-annealed states (Figure 20).

**Figure 21:**
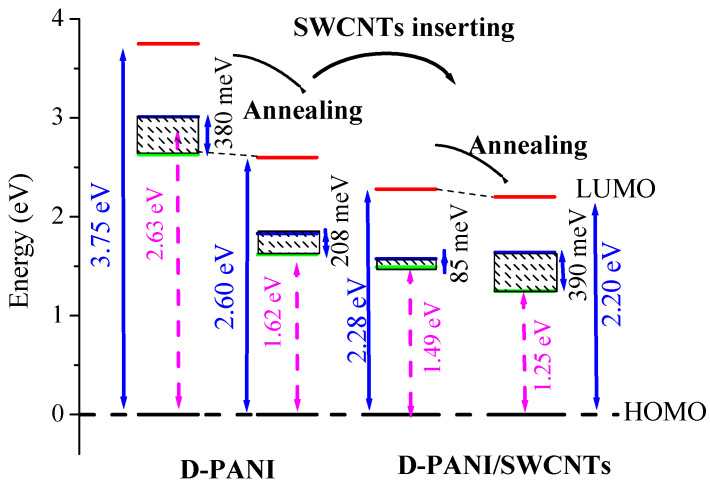
Change in optical parameters under doping and annealing.

On the red side of the spectra, annealing improves the extinction coefficient. For the doped state of PANI, thermal treatment leads to a change in the oxidation degree and localized states can overlap with the LUMO level. The extinction coefficient may be improved as a consequence of the new energetic configuration. For the nanocomposite, a net amelioration in the spectral range from 600 to 850 nm is observed after annealing. Indeed, good SWCNT dispersion leads to a covalent bonding, as previously reported [46]. If the overlapping between diffusion length and nanoparticles repartition is satisfied, charge transfer from the doped matrix to CNTs can be established. From Tauc formalism, the calculation of band width of the corresponding localized states (E_U_) demonstrates the decrease in the latter when annealing, probably due to the already discussed self-cross-linking effects [133,134,143]. Figure 21 represents all changes in the electronic structure after annealing and adding CNTs.

For the nanocomposite, the E_U_ (localized state band width) is nearly 85 meV and 390 eV for not-annealed and annealed states, respectively, involving a charge transfer from the organic matrix (self-cross-linked) to CNTs, as previously reported [21]. The large value after annealing is the consequence of well-dispersed nanoparticles, involving good compatibility between the diffusion length and CNT repartition. A microscopic study mentions that bundled CNTs before annealing became relatively dispersed and homogeneously distributed, with the repartition size varying from 3 to 8 nm [132]. At the molecular scale, annealing leads to partially dispersed and oriented SWCNTs in cross-linked PANI. Electron–hole pairs, which constitute the exciton pairs, are born in the PANI matrix and then migrate by diffusion [20,144]. From the Scharber diagram, the resulting electronic structure presents a band gap suitable for a relatively higher conversion efficiency and is qualified by good compatibility with the solar spectrum [145].

## 7. Conclusions

A study of the structural, optical, and electronic properties of diverse polymers containing nitrogen atoms was performed. Some parameters are fundamental to achieving better dispersion of CNTs in the organic matrix, namely the choice of the solvent and thermal treatment. Short oligo-N-vinylcarbazole can be spontaneously functionalized with SWCNTs when chloroform is the medium. However, moderate thermal treatment is needed when chlorobenzene is the medium. Though the complete isolation of SWNTs can be achieved, chlorobenzene is more efficient in the SWCNT dispersion process. Correlation of experimental and theoretical study (DFT) in both cases demonstrates that there is a grafting of vinylidene groups to the nanotube side wall. For PVK-P3HT, a good dispersion state is obtained even at a higher SWCNT concentration, reaching 60% of CNTs, and the grafting is limited to the hexylthiophene sequences. The conservation of the hexylthiophene molecular structure leads to excellent properties for photovoltaic conversion, such as compatibility with the solar spectrum and the effectiveness of the dynamics of the excited states.

PANI itself is an insulating material, because of which its functionalization with CNTs was preceded by acid doping. Without thermal treatment, PANI is linked to SWCNTs via nitrogen atoms, leading to some structural and optical changes. A decrease in the optical gap of 2.28 eV gives rise to good compatibility with the solar spectrum. Moreover, donor–acceptor charge transfer creates a localized state within the band gap, with an onset energy of 1.62 eV. The modeling of a prototype bulk hetero-nanojunction photovoltaic device gives a typical open-circuit voltage of 1.25 V and a PCE of 4~5%. Annealing induces the self-cross-linking of PANI in its doped state and the dispersion of CNTs in the organic matrix. Otherwise, thermal treatment influences the electronic structure, leading to a band gap of 2.20 eV and localized states having a bandwidth of 390 meV. Moreover, it was found that skin depth and extinction coefficient variations are closely related to a molecular arrangement induced by annealing, namely self-cross-linking. As a consequence of the number of oxidation degrees of PANI, the corresponding interpenetrating network (doped PANI/CNTs) exhibits excellent compatibility with the solar spectrum and shows good coherence with the diffusion length, making it a good candidate for the active layer in organic photovoltaic devices.

As a consequence of the above-mentioned structural changes, adding CNTs to the polymer matrix leads to a broader absorption spectrum, PL quenching, and a shorter excited lifetime. In fact, these optical criteria are coherent with charge transfer from the organic matrix to the CNTs due to the interpenetrating P–N nanojunctions.

## Figures and Tables

**Table 1:** Decay parameters of OVK/SWCNT nanocomposites [18].

Samples	P_1_ (%)	P_2_ (%)	τ_1_ (ns)	τ_2_ (ns)	A_1_	A_2_	τ_m_ (ns)
OVK	18.8	81.10	0.983	6.223	8.911	6.042	5.23
OVK/SWCNTs in chloroform	32.73	67.26	0.753	6.996	15.575	3.445	4.95
OVK/SWCNTs in chlorobenzene	32.98	67.01	0.662	3.848	13.747	4.804	2.796

## Data Availability

The data supporting the reported results of this study will be made available by the authors upon request.
